# Evaluation of Different Breast Cancer Screening Strategies for High-Risk Women in Beijing, China: A Real-World Population-Based Study

**DOI:** 10.3389/fonc.2021.776848

**Published:** 2021-11-04

**Authors:** Xi Zhang, Lei Yang, Shuo Liu, Huichao Li, Qingyu Li, Yangyang Cheng, Ning Wang, Jiafu Ji

**Affiliations:** ^1^ Key Laboratory of Carcinogenesis and Translational Research (Ministry of Education/Beijing), Beijing Office for Cancer Prevention and Control, Peking University Cancer Hospital & Institute, Beijing, China; ^2^ Key Laboratory of Carcinogenesis and Translational Research (Ministry of Education/Beijing), Gastrointestinal Cancer Center, Peking University Cancer Hospital and Institute, Beijing, China

**Keywords:** participation rate, breast cancer screening, diagnostic performance, real-world study, Chinese women

## Abstract

**Background:**

Mammography-based breast cancer screening has been widely implemented in many developed countries. Evidence was needed on participation and diagnostic performance of population-based breast cancer screening using ultrasound in China.

**Methods:**

We used data from the Cancer Screening Program in Urban China in Beijing from 2014 to 2019 and was followed up until July 2020 by matching with the Beijing Cancer Registry database. Eligible women between the ages of 45 and 69 years were recruited from six districts and assessed their risk of breast cancer through an established risk scoring system. Women evaluated to be at high risk of breast cancer were invited to undergo both ultrasound and mammography. Participation rates were calculated, and their associated factors were explored. In addition, the performance of five different breast cancer screening modalities was evaluated in this study.

**Results:**

A total of 49,161 eligible women were recruited in this study. Among them, 15,550 women were assessed as high risk for breast cancer, and 7,500 women underwent ultrasound and/or mammography as recommended, with a participation rate of 48.2%. The sensitivity of mammography alone, ultrasound alone, combined of ultrasound and mammography, ultrasound for primary screening followed by mammography for triage, and mammography for preliminary screening followed by ultrasound for triage were19.2%, 38.5%, 50.0%, 46.2%, and 19.2%, and the specificity were 96.1%, 98.6%, 94.7%, 97.6%, 95.7%, respectively. The sensitivity of combined ultrasound and mammography, ultrasound for primary screening followed by mammography for triage, was significantly higher than mammography alone (p=0.008 and p=0.039). Additionally, ultrasound alone (48,323 RMB ($7,550)) and ultrasound for primary screening followed by mammography for triage (55,927 RMB ($8,739)) were the most cost-effective methods for breast cancer screening than other modalities.

**Conclusions:**

Ultrasound alone and ultrasound for primary screening and mammography are superior to mammography for breast cancer screening in high-risk Chinese women.

## Introduction

Breast cancer has surpassed lung cancer as the most commonly diagnosed cancer worldwide, with an estimated 2.3 million new cases occurred in 2020 (1). In China, the incidence of breast cancer has increased by 3-5% annually for the past twenty years, twice as faster as the global rate (2). Due to the limitation of health resources and the lack of a comprehensive national breast cancer screening program, the vast majority of breast cancer patients in China are diagnosed at a late stage, resulting in a high proportion of poor prognosis (2, 3). A pooled analysis of cancer registry data has revealed that the age-standardized 5-year relative survival rate for breast cancer among Chinese women is 82.0% in 2012-2015 (4), which was significantly lower than that in Western countries such as the United States (91.1% in 2007-2013) (5).

Sufficient randomized controlled trials (RCTs) have confirmed that mammography-based breast cancer screening can effectively reduce breast cancer mortality in women aged 50-69 (6). Therefore, mammography has been widely adopted in national breast cancer screening programs in many developed countries; however, its performance declined dramatically among women with dense breast tissue and young women (7–9). It is noteworthy that women with extremely dense breast tissue have an increased risk of breast cancer, and more youthful breast cancer patients contributed a much heavier disease burden than elderly patients. Like other Asian women, Chinese women tend to have smaller and denser breasts than Western women (10). Furthermore, the peak onset age at breast cancer diagnosis was about 40 to 50 years in Chinese women, approximately ten years younger than in Western countries (11). Therefore, screening with mammography alone may not be the best option for breast cancer screening in China.

Ultrasound is a potential method to improve breast cancer detection, especially in women with dense breast tissue and younger women. Some Western countries recommended ultrasound as the supplementary of mammography because ultrasound is non-invasive, non-radioactive, and more appropriate for smaller and denser breasts. Although some studies conducted in Japan and China have used ultrasound as the primary tool for breast cancer screening (12, 13), these studies have undergone strict quality control and did not follow the health outcomes. Thus, the real-world performance of ultrasound and mammography for breast cancer screening in such women is urgent.

In 2012, the government of Beijing launched a registered organized population-based cancer screening program (The Beijing Cancer Screening Prospective Cohort Study, BCSPCS) to screen common cancers, including breast cancer. For the present study, we reported the breast cancer screening results in Beijing between 2014 and 2019. The purpose of this study was to evaluate the participation rate and diagnostic yield of ultrasound and mammography for screening breast cancer, as well as to explore a cost-effective breast cancer screening strategy for high-risk Chinese women.

## Materials and Methods

### Study Design and Participants

**Figure 1:**
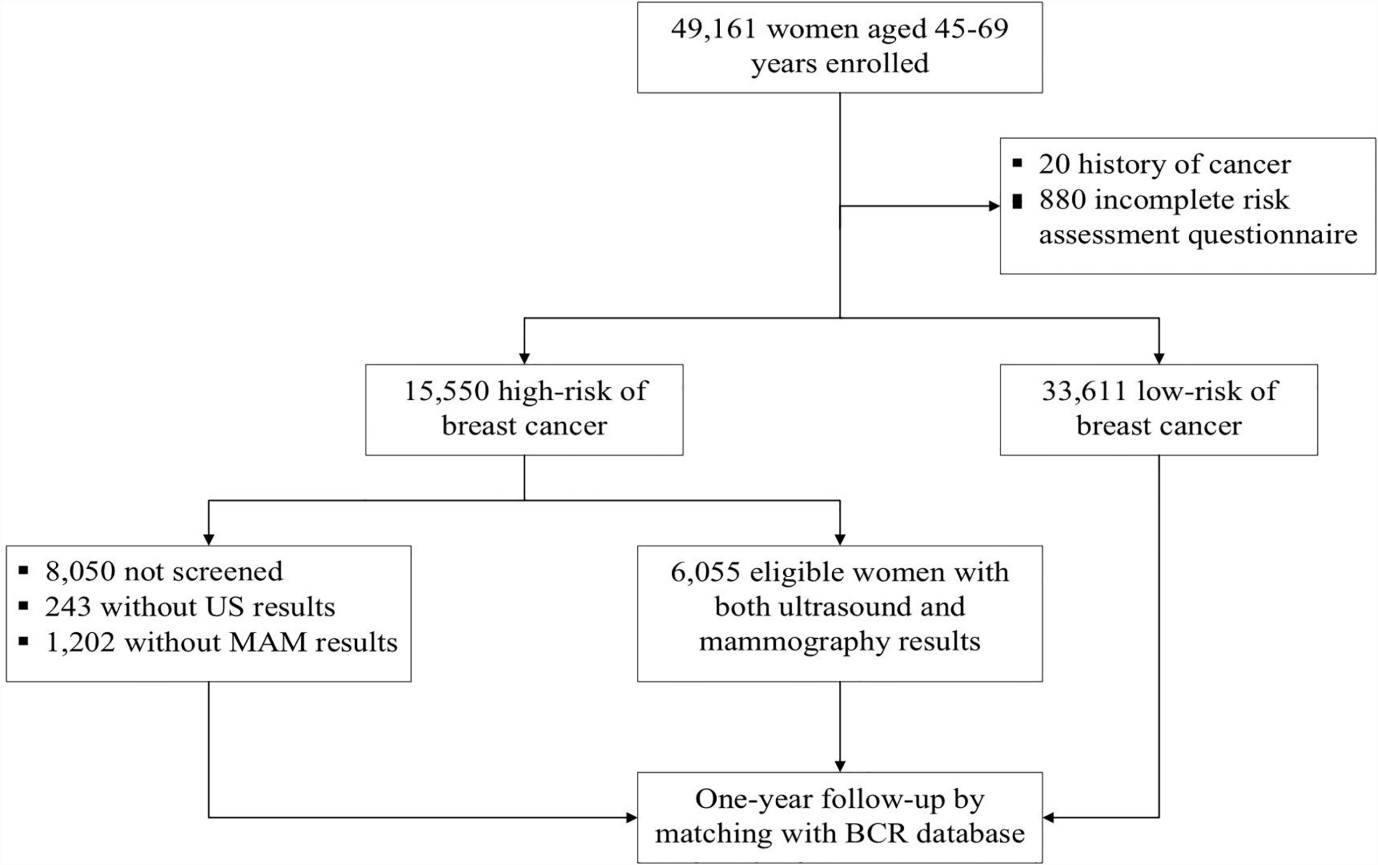
Study flowchart of participant’s enrollment, screening and follow-up of breast cancer screening in BCSPCS, 2014-2019.

We performed a population-based prospective breast cancer screening program among female residents in Beijing from 2014 to 2019. This study was conducted under the framework of the Cancer Screening Program in Urban China (CanSPUC). In brief, asymptomatic females living in the designated communities of the six participating districts were approached by trained staff through personal encounters or phone calls. Social media and community advertisements were used to raise public awareness of this cancer screening program. Eligible women were aged 45-69 years without a history of any cancer and registered in Beijing for at least three years were enrolled in this study. After signing the written informed consent, all eligible women were interviewed one-on-one by a trained community healthcare personnel to collect their socio-demographic characteristics and potential breast cancer risk factors. Next, an established risk scoring system was used to evaluate their risk of developing breast cancer. After that, women identified as high-risk of breast cancer were invited to undergo free ultrasound and mammography examinations at the tertiary-level hospital designated by the program. One year later, all subjects were followed up passively by matching the Beijing Cancer Registry (BCR) database to obtain their health outcome information (whether they were diagnosed with breast cancer or not). The Ethics Committee of National Cancer Center/Cancer Hospital Chinese Academy of Medical Sciences and Peking Union Medical College approved the study and written informed consent was obtained from all participants prior to implementation. A flowchart of the study is shown in **Figure 1**.

### Risk Assessment

All participants were invited to health facilities and completed a paper-based questionnaire to collect individual breast cancer exposure information. Then, health professionals used an online tool to measure the personal risk of breast cancer which was followed the Harvard Risk Index (14), but the included risk factors, relative risks, and exposure rates of risk factors were adjusted according to the characteristics of the Chinese women. The following factors were included in the risk scoring system of breast cancer: age, body mass index (BMI), age of menarche, menopause status, age of first marriage, age at first delivery, total months of breastfeeding, history of benign breast diseases, history of female reproductive system surgery, and family history of breast cancer. Each risk factor was given a coefficient score by the expert panel based on the magnitude of its association with breast cancer. The cumulative risk scores were calculated and were then divided by the average risk score in the general population to get the final individual relative risks. Women with a relative risk of 1.50 or higher were considered at high risk for breast cancer.

### Clinical Procedure

Women at high risk for breast cancer were invited to receive a clinical breast examination performed by physicians in the tertiary-level hospital, followed by successive ultrasound and mammography conducted by experienced radiologists with at least five years of experience. Any abnormal findings during the examination were carefully checked and were required to be photo-documented. In addition, clinical information such as morphology, breast density, the structure of the gland, tumor characteristics (position, size, margin, echogenicity, etc.), and the characterizations of lesions was recorded. All images of ultrasound and mammography were stored and transferred to the research center for subsequent verification at the end of the annual project.

According to the Breast Imaging Reporting and Data System (BI-RADS) classification system, images of either type of imaging investigation were interpreted and classified into the following five categories (15): 1, negative; 2, benign; 3, probably benign; 4, suspicious; and 5, highly suggestive of malignancy. Images were interpreted by two qualified radiologists with at least five years of working experience at each hospital. Consideration of physicians’ sufficient qualification, reports of ultrasound and mammography were issued by each doctor independently. For inconsistent cases, the radiologists reached a collaborative agreement through discussion. Women identified as BI-RADS category 3 were recommended for six months follow-up or additional imaging. Women identified as category 4 or 5 were recommended for biopsy and underwent necessary treatment. All procedures strictly followed the clinical routine and were shown in **Figure 1**.

### Data Collection and Quality Control

This study had three documents, including a risk assessment questionnaire, ultrasound examination form, and mammography examination form, all of which were filled out by trained healthcare workers or physicians. Then, data entry clerks at each community were employed to input the data from the paper documents to the online data management system. After that, researchers downloaded all the original data, performed logical verification of the data quality, and performed further analysis.

### Follow-up Data

All participants with breast cancer risk assessment results were passively followed up by matching their identification number with the BCR database to obtain their health outcomes at one-year intervals from October 1, 2014, to July 31, 2020. BRC has high standards of data accuracy population-based cancer registry covering 13 million (nearly 100%) permanent residents in Beijing (16). Besides, all breast cancer cases were classified by sites according to the International Statistical Classification of Disease and Related Health Problems Tenth Revision (ICD-10). Furthermore, breast invasive carcinoma (ICD-10: C50.0-C50.9) and breast carcinoma *in situ* (ICD-10: D05.0-D05.9) were extracted and analyzed from the whole cancer database.

### Cost Analysis

The cost per breast cancer detected was calculated by dividing the total cost of a screening program by the number of breast cancer detected. The program cost included consultation, physician exam, ultrasound and/or mammography exam, biopsy, and histopathology. In this study, we used the estimated cost of China’s ‘Two Cancer (breast cancer and cervical cancer) Screening’ campaign, which was 5 RMB (Chinese Yuan) for consultation and physical exam, 70 RMB for ultrasound, 200 RMB for mammography, and 300 RMB for biopsy (Ministry of Health of China and All-China Women’s Federation, 2009). We used the exchange rate of 6.40:1 to convert RMB to the US dollar.

### Statistical Analysis

This study took the individual woman as the unit of analysis. The socio-demographic characteristics of the participants were described by mean and standard deviations (SD) of continuous variables or proportion and percentages of categorical variables. The chi-square test was used to compare the difference in participation rates between each group. Univariate logistic regression was employed to analyze the association of potential factors with examination participation rates. The parameters that were found to be significant (p <0.01) by univariate analysis were incorporated and examined using multivariate analysis. Odds ratios (ORs) with corresponding 95% confidence intervals (95% CIs) were calculated and reported according to the Wald chi-square statistics. Diagnostic yield was calculated for both screening and non-screening groups, including the histologic type and stages of breast cancer. One-year follow-up results of the cancer registry were regarded as the ‘gold standard’ of breast cancer screening. Sensitivity and specificity were calculated and compared with McNemar’s test. The area under the curve (AUC) of the receiver operating characteristics (ROC) and their 95% CIs of different screening modalities were assessed and compared using the *Z* test. The above-mentioned statistical analyses were conducted with the use of SAS software, version 9.4 (Cary, NC, USA), and the statistical significance level was set at 0.05 for two-sided tests.

## Results

### Participant Characteristics

From October 2014 to July 2019, 49,161 eligible women were recruited from the designated communities in BCSPCS and completed the risk assessment questionnaire. After excluding 33,611 who were assessed as low risk of breast cancer, the remaining 15,550 women were identified as being at high risk of developing breast cancer and were invited to attend both ultrasound and mammography examination. The characteristic of the high-risk population and women who completed ultrasound and/or mammography were presented in **Table 1**. Overall, participants were predominantly (69.3%) between 50 and 64 years old, with a mean age of 56.7 years (SD=6.5 years) for the high-risk population. In addition, most high-risk women were married, postmenopausal, and had their first child before 28 years old. Among them, 11.1% were current smokers, 60.0% had a history of breastfeeding, 77.9% had a history of benign breast diseases, and 36.0% reported a family history of breast cancer. More demographic characteristics of the study population are summarized in **Table 1**.

**Table 1:** Characteristics of the women and participation rates.

Characteristics	At high risk of breast cancer, n (%)	Underwent US and/or MG, n (%)	Participation rate, %	*χ^2^*	*P* value
Age, years				18.62	0.001
45-49	2634 (16.9)	1260 (16.8)	47.8		
50-54	3353 (21.6)	1678 (22.4)	50.0		
55-59	3913 (25.2)	1907 (25.4)	48.7		
60-64	3509 (22.6)	1707 (22.8)	48.6		
65-69	2141 (13.8)	948 (12.6)	44.3		
Marriage				20.68	<0.001
Single, divorced, widowed	2434 (15.7)	1071 (14.3)	44.0		
Married	13116 (84.3)	6429 (85.7)	49.0		
BMI, kg/m^2^				21.53	<0.001
<18.5	288 (1.9)	125 (1.7)	43.4		
18.5-24.0	6191 (39.8)	2864 (38.2)	46.3		
24.0-28.0	6182 (39.8)	3097 (41.3)	50.1		
≥28.0	2889 (18.6)	1414 (18.9)	48.9		
Education, years					
≤9	4471 (28.8)	2157 (28.8)	48.2	0.08	0.963
10-12	6183 (39.8)	2989 (39.9)	48.3		
≥13	4896 (31.5)	2354 (31.4)	48.1		
Smoking status				16.57	<0.001
Never or rarely	13364 (85.9)	6367 (84.9)	47.6		
Current	1724 (11.1)	876 (11.7)	50.8		
Former	462 (3.0)	257 (3.4)	55.6		
Age at menarche, years				0.26	0.609
<12	835 (5.4)	410 (5.5)	49.1		
≥12	14708 (94.6)	7088 (94.5)	48.2		
Menopause status				16.70	<0.001
Premenopausal	4380 (28.2)	1998 (26.6)	45.6		
Postmenopausal	11170 (71.8)	5502 (73.4)	49.3		
Age at first live birth, years				1.18	0.277
<28	8448 (66.0)	4358 (66.4)	51.6		
≥28	4358 (34.0)	2204 (33.6)	50.6		
Breast feeding				115.02	<0.001
Yes	8761 (60.0)	4574 (64.5)	52.2		
No	5844 (40.0)	2522 (35.5)	43.2		
History of benign breast diseases				311.50	<0.001
Yes	12107 (77.9)	6296 (83.9)	52.0		
No	3443 (22.1)	1204 (16.1)	35.0		
Family history of breast cancer				213.15	<0.001
Yes	5404 (36.0)	3031 (41.9)	56.1		
No	9612 (64.0)	4199 (58.1)	43.7		

BMI, body mass index (calculated as weight (kg)/height (m) ^2^); MG, mammography; US, ultrasound.

### The Participation Rate for Breast Cancer Screening and Its Associated Factors

Among the 15,550 women at high risk of breast cancer, 7,500 received ultrasound and/or mammography, and the participation rate was 48.2% in our study. Overall, women aged 50-64 years had a higher participation rate than other age groups. Besides, univariate analyses showed that women who were married, had a BMI higher than 18.5, currently or had smoked, postmenopausal, had a history of breastfeeding, benign breast diseases, and a family history of breast cancer had relatively higher participation rates compared to the other groups (p<0.05). We also calculated multivariate logistic regression models to explore the potential factors associated with participation rates, as shown in **Table 2**. We found that age, BMI, menopause status, breastfeeding history, history of benign breast diseases, and family history of breast cancer were associated with participation rate (p<0.01). Specifically, the odds of postmenopausal women, women with a history of breastfeeding, benign breast diseases, and a family history of breast cancer underwent ultrasound and/or mammography were 23% (aOR=1.23, 95% CI=1.11-1.37), 27% (aOR=1.27, 95% CI=1.18-1.36), 95% (aOR=1.95, 95% CI=1.78-2.14) and 49% (aOR=1.49, 95% CI=1.39-1.59) higher than those women in other groups.

**Table 2:** Factors associated with participation rate in breast cancer screening in Beijing.

Factors	*OR* (95% *CI*)	*P* value	a*OR* ^a^ (95% *CI*)	*P* value
Age, years		0.001		<0.001
45-49	1.00		1.00	
50-54	1.09 (0.99-1.21)	0.090	0.98 (0.87-1.11)	0.764
55-59	1.04 (0.94-1.14)	0.475	0.86 (0.75-0.98)	0.022
60-64	1.03 (0.93-1.14)	0.529	0.85 (0.74-0.98)	0.023
65-69	0.87 (0.77-0.97)	0.014	0.76 (0.65-0.88)	<0.001
Marriage				
Single, divorced, widowed	1.00	<0.001		
Married	1.22 (1.12-1.34)			
BMI, kg/m^2^		<0.001		0.004
<18.5	1.00		1.00	
18.5-24.0	1.12 (0.89-1.43)	0.342	0.99 (0.77-1.27)	0.932
24.0-28.0	1.31 (1.03-1.66)	0.027	1.12 (0.87-1.44)	0.379
≥28.0	1.25 (0.98-1.60)	0.073	1.27 (1.18-1.36)	0.371
Smoking status		<0.001		
Never or rarely	1.00			
Current	1.14 (1.03-1.26)	0.013		
Former	1.38 (1.14-1.66)	0.001		
Menopause status				
Premenopausal	1.00		1.00	<0.001
Postmenopausal	1.16 (1.08-1.24)	<0.001	1.23 (1.11-1.37)	
Breast feeding				
No	1.00		1.00	<0.001
Yes	1.44 (1.35-1.54)	<0.001	1.27 (1.18-1.36)	
History of benign breast diseases				
No	1.00		1.00	<0.001
Yes	2.02 (1.86-2.18)	<0.001	1.95 (1.78-2.14)	
Family history of breast cancer				
No	1.00		1.00	<0.001
Yes	1.65 (1.54-1.76)	<0.001	1.49 (1.39-1.59)	

BMI, body mass index (calculated as weight (kg)/height (m) ^2^); CI, confidence interval; OR, odds ratio.

a Odds ratios were adjusted for factors including age, marriage status, body mass index, smoking status, menopause status; history of breast feeding, benign breast diseases; and family history of breast cancer in the logistic regression models.

### Histopathology Results in Screening and Non-Screening Groups

A total of 105 breast cancer were identified by matching the BCR database, with overall incidence rates of 213.6/100,000 (105/49,161). The incidence rate of breast cancers was 172.6/100,000 (58/33,611) in the low-risk group and 302.3/100,000 (47/15,550) in the high-risk group, respectively. Of the 7,500 women who underwent the screening, 30 breast cancers were diagnosed, including 21 with invasive breast cancer and 9 with ductal carcinoma *in situ* (DCIS). Among the screening group, 14 cases (11 with invasive breast cancer and 3 with DCIS) were detected, with a detection rate of 46.7% (14/30) and an early diagnostic rate of 77.8% (7/9). However, 16 breast cancers (10 with invasive breast cancer and 6 with DCIS) were undetected in our study, of which 88.9% (8/9) were early cases, and the missed diagnosis rate was 53.3% (16/30). For 8,050 women who were at high risk of breast cancer but did not receive any examination, 17 breast cancers (13 with invasive breast cancer and 4 with DCIS) were diagnosed, 71.4% (5/7) were early-stage cases. Furthermore, among the 33,611 women with a low risk of breast cancer, 58 breast cancer were found, of which early cases accounted for 30.4% (7/23). More characteristics of detected and undetected breast cancer are summarized in **Table 3**.

**Table 3:** Characteristics of detected and undetected breast cancer, n (%).

Characteristics	Low-risk of breast cancer (n = 33,611)	High risk of breast cancer (n = 15,550)			
		No screening (n = 8,050)	Screening (n = 7,500)		
			Screened	Detected	Undetected
Histological type					
Invasive breast cancer	51 (0.15)	13 (0.16)	21 (0.28)	11 (0.15)	10 (0.13)
DCIS	7 (0.02)	4 (0.05)	9 (0.12)	3 (0.04)	6 (0.08)
Total	58 (0.17)	17 (0.21)	30 (0.40)	14 (0.19)	16 (0.21)
Having TNM stage	23	7	18	9	9
Early-stage cases (rates)	7 (30.4)	5 (71.4)	15 (83.3)	7 (77.8)	8 (88.9)

DCIS, ductal carcinoma in situ; TNM, Tumor, Node, Metastasis.

### Diagnostic Performance and Cost of Different Breast Cancer Screening Modalities

We compared the performance of five different breast cancer screening modalities in this study, including mammography alone (model 1), ultrasound alone (model 2), combined of ultrasound and mammography (model 3), ultrasound for primary screening followed by mammography for triage (model 4), and mammography for primary screening followed by ultrasound for triage (model 5). As shown in **Table 4**, the sensitivities of the above five screening modalities were 19.2%, 38.5%, 50.0%, 46.2%, and 19.2%, and the specificity were 96.1%, 98.6%, 94.7%, 97.6%, 95.7%, respectively. McNemar’s test shown the sensitivity of model 3 and model 4 was significantly higher than that of mammography alone (p=0.008 and p=0.039). The specificity of model 2 and model 4 was significantly higher than that of mammography alone, while model 3 and model 5 were lower than that of mammography alone (p<0.001). Moreover, there was a significant difference in AUC (p<0.001) among model 3, model 4 and using mammography alone.

**Table 4:** Comparison of performance of different breast cancer screening modalities among Chinese women.

	Model 1	Model 2	Model 3	Model 4	Model 5
Sensitivity, % (95% CI)	19.23 (6.55-39.35)	38.46 (20.23-59.43)	50.00 (29.93-70.07)*	46.15 (26.59-66.63)*	19.23 (6.55-39.35)
Specificity, % (95% CI)	96.09 (95.56-96.56)	98.56 (98.22-98.84)*	94.73 (94.13-95.28)*	97.61 (97.19-97.98)*	95.65 (95.11-96.16)*
AUC	0.58 (0.56-0.59)	0.69 (0.67-0.70)	0.72 (0.71-0.73)*	0.72 (0.71-0.73)*	0.57 (0.56-0.59)*
**Cost analysis**					
Physical exam	5 RMB × 6055	5 RMB × 6055	5 RMB × 6055	5 RMB × 6055	5 RMB × 6055
Mammography	200 RMB × 6055	0	200 RMB × 6055	200 RMB × 851	200 RMB × 6055
Ultrasound	0	70 RMB × 6055	70 RMB × 6055	70 RMB × 6055	70 RMB × 1077
Biopsy	300 RMB × 241	300 RMB × 97	300 RMB × 331	300 RMB × 156	300 RMB × 267
Total cost (RMB)	1313575	483225	1764425	671125	1396765
No. of detected breast cancer	5	10	13	12	5
Cost per breast cancer, RMB	262715	48323	135725	55927	279353
Cost per breast cancer, US dollar	41049	7550	21207	8739	43649

AUC, the area under the receiver operating characteristic curve; CI, confidence interval.

Model 1: Mammography alone; Model 2: Ultrasound alone; Model 3: Ultrasound and Mammography co-testing; Model 4: Ultrasound BI-RADS 3 then Mammography; Model 5: Mammography BI-RADS 3 then Ultrasound; 1.00 US dollar=6.40 RMB (Chinese Yuan).

*The different in the estimate between using mammography alone (model 1) and other screening models was statistically significant.

Regarding the cost of the screening, we found that the most cost-effective method for finding each breast cancer was ultrasound alone (48,323 RMB ($7,550)) and ultrasound for primary screening followed by mammography for triage (55,927 RMB ($8,739)), followed by combined of ultrasound and mammography (135,725 RMB ($21,207)). The worst was mammography alone (262,715 RMB ($41,049)) and mammography for primary screening followed by ultrasound for triage (279,353 RMB ($43,649)).

## Discussion

We reported the results of 49,161 women participating in breast cancer screening in urban areas of China. To the best of our knowledge, this is the first large-scale population-based prospective cohort study of breast cancer screening in high-risk women in China. We achieved the participation rates and the detection rates of breast cancer screening in different populations and reported five screening modalities’ diagnostic yield and cost-effectiveness. These results suggest that various screening schemes targeting specific people are needed and provide evidence support for improving the effectiveness of screening in the future.

Despite sufficient scientific evidence to support that screening reduces breast cancer mortality, the participation rate of high-risk women in our study has remained lower (48.2%) than organized breast cancer screening programs conducted in other countries and regions (50%-80%) (17–20). In addition, we identified several factors, including age, BMI, menopause status, breastfeeding, history of benign breast diseases, and family history of breast cancer, potentially associated with the participation rate. The reasons for dropout are complex, which may affect by personal, socioeconomic, and cultural factors. In this study, psychological factors also contributed to the low participation rate. Many women mistakenly believed that they would not receive adequate treatment after being diagnosed with breast cancer, which would bring a substantial financial and psychological burden. As a result, they refused to be screened for breast cancer. This result suggests that future breast cancer screening in China will combine the risk stratification mechanism with public education and motivation to improve the low compliance of selected high-risk populations.

Of the 7,550 women who underwent ultrasound or mammography examination, 14 breast cancers were detected, with a detection rate of 1.87/1,000. Another study conducted in urban China showed that women who received ultrasound and mammography screening had a breast cancer detection rate of 0.56/1,000 after one year of follow-up, which was much lower than the current study (21). It may be due to the young age of the women included for screening in the above research and the high rate of missed visits in that population. Moreover, in the present study, 83.3% of the cancers found by cancer screening were in stages 0 and I, which were probably curable by surgery alone. However, in the no screening group, only 71.4% were early-stage cases, suggesting that screening was effective.

Our study demonstrates that ultrasound performed better or at least not worse than mammography in real-world settings, with higher sensitivity and specificity in high-risk Chinese women, as has been consistently shown in previous studies (13, 22). However, it is contrary to screening performed in Western countries (7, 23). We attribute the low sensitivity of mammography to two main reasons. One is that dense breast tissue decreases the sensitivity of mammography, resulting in nearly one-third of breast cancers going undetected (24). The underlying dense breast tissue can obscure the radiological features of early breast cancer. Chinese women characteristically have higher-density breasts than women from other ethnic groups, with more than half of Chinese women aged 45-65 years categorized as having dense breasts (10). As a result, high accuracy is difficult to achieve with mammography screening alone. On the other hand, the mean age at breast cancer diagnosis was 59.3 years in this study, while mammography performed less sensitively in younger patients (25–27). However, it should be noted that ultrasound is operator-dependent and has poor repeatability, constraining its use in developing countries. A study found that the sensitivity values for the experienced readers and inexperienced readers were 91.7% and 66.7%, respectively (28). Recently, researchers have been paying considerable attention to the automated breast ultrasound system (ABUS), which has also been named three-dimensional ultrasonography (22, 29–31). Unlike traditional hand-held ultrasound, ABUS has a reproducible and less operator-dependent process for imaging acquisition and image interpretation. It allows radiologists to review the entire dataset and interpret the images remotely through the Cloud, improving reproducibility and decrease variability. Thus, ABUS might be a promising technology to replace traditional hand-held ultrasound for breast cancer screening.

Cost-effectiveness is one of the key elements for large-scale population screening. As far as we know, in the United States and other Western countries, ultrasound is expensive and usually not covered by insurance for breast cancer screening (32, 33). However, in China, the cost of the ultrasound examination is only one-third of that of mammography, and ultrasound is widely available and relatively inexpensive in China, even in remote and low-resource areas. In this present study, ultrasound for breast cancer screening is the lowest per-cancer-finding cost, which is only 18.4% of mammography or 35.6% of combined methods. Besides, using ultrasound for primary screening and mammography for triage was equally cost-effective as ultrasound. Currently, China’s economic level varies widely among regions. The areas along the east coast in China are relatively developed, while those in the west are comparatively underdeveloped. Due to this, a large-scale breast cancer screening program might be conducted using different screening strategies in China, depending on various economic conditions. To be specific, in remote and resource-limited areas, ultrasound alone could be used for breast cancer screening, while in developed urban areas, a screening model using ultrasound for primary screening and mammography triage could be considered.

Notably, our study found that the overall breast cancer detection rate was only slightly higher in the screening group than in the non-screening group. Given the relatively low participation rate in screening, some breast cancer cases were missed during the program, which substantially reduced the effectiveness of screening. To improve the diagnostic yield of breast cancer screening in China, the following issues should be further addressed. First, optimize the risk assessment score based on current research findings and proven risk prediction scores, and incorporate genetic testing into risk assessment models in available regions. Second, design novel breast cancer screening strategies suitable for women in different areas and at different risks of breast cancer. Third, carry out multifactor interventions targeting multiple levels of care to optimize breast cancer screening compliance.

The findings in this study need to be considered in light of its limitations. Firstly, data quality depends largely on the experience of the radiologists and healthcare staff who interviewed the participant. Thus, the results of Beijing cannot be generalized to other regions in China. Secondly, we used a self-reported questionnaire to assess the risk factor of breast cancer, so some residents may provide untrue information about breast cancer for the sake of getting free examinations. Thirdly, we used the cancer registry data as the endpoint of this study. However, the cancer registration data had about half-years later than the diagnostic time at the hospital that would miss some patients. Moreover, although Beijing had a complete registration system, there were still a small number of patients that may be omitted. Therefore, the sensitivity of this study may be overestimated, and the specificity may be underestimated. Fourthly, the current registration system does not require reporting of cancer stage, so there may be errors in the staging in this study. Last but not least, this study was a real-world screening program rather than well-designed scientific research; therefore, we did not collect the data on recall rates, the number of pathological examinations, and the number of benign cases. In addition, the small sample size of this study also resulted in limited applicability and worth of the findings.

## Conclusions

In conclusion, ultrasound alone or ultrasound for primary screening and mammography for triage offers a sensitive and cost-effective way for breast cancer screening in high-risk Chinese women. Long-term follow-up is needed to assess whether these approaches could reduce advanced breast cancer and breast cancer mortality in the future.

## Data Availability Statement

The original contributions presented in the study are included in the article/supplementary material. Further inquiries can be directed to the corresponding authors.

## Ethics Statement

The studies involving human participants were reviewed and approved by National Cancer Center/Cancer Hospital Chinese Academy of Medical Sciences and Peking Union Medical College. The patients/participants provided their written informed consent to participate in this study.

## Author Contributions

JJ, NW, and LY developed the protocol for the study. HL, QL, and YC executed and coordinated this study. XZ performed the statistical analyses and drafted the manuscript. SL participated in the data collection and revised the manuscript. All authors read and approved the final version of the manuscript.

## Funding

This study was funded by Beijing excellent talents training project (No. 2016000021469G189). The funder did not participate in any part of the study from study design to approval of the manuscript, except for supporting this project.

## Conflict of Interest

The authors declare that the research was conducted in the absence of any commercial or financial relationships that could be construed as a potential conflict of interest.

## Publisher’s Note

All claims expressed in this article are solely those of the authors and do not necessarily represent those of their affiliated organizations, or those of the publisher, the editors and the reviewers. Any product that may be evaluated in this article, or claim that may be made by its manufacturer, is not guaranteed or endorsed by the publisher.
